# Building Open Access to Research (OAR) Data Infrastructure at NIST

**DOI:** 10.5334/dsj-2019-030

**Published:** 2019

**Authors:** Gretchen Greene, Raymond Plante, Robert Hanisch

**Affiliations:** National Institute of Standards and Technology, Material Measurement Laboratory Office of Data and Informatics, Gaithersburg, Maryland, US

**Keywords:** Data Repository, FAIR, research metadata, metrology, data portal, government

## Abstract

As a National Metrology Institute (NMI), the USA National Institute of Standards and Technology (NIST) scientists, engineers and technology experts conduct research across a full spectrum of physical science domains. NIST is a non-regulatory agency within the U.S. Department of Commerce with a mission to promote U.S. innovation and industrial competitiveness by advancing measurement science, standards, and technology in ways that enhance economic security and improve our quality of life. NIST research results in the production and distribution of standard reference materials, calibration services, and datasets. These are generated from a wide range of complex laboratory instrumentation, expert analyses, and calibration processes. In response to a government open data policy, and in collaboration with the broader research community, NIST has developed a federated Open Access to Research (OAR) scientific data infrastructure aligned with FAIR (Findable, Accessible, Interoperable, Reusable) data principles. Through the OAR initiatives, NIST’s Material Measurement Laboratory Office of Data and Informatics (ODI) recently released a new scientific data discovery portal and public data repository. These science-oriented applications provide dissemination and public access for data from across the broad spectrum of NIST research disciplines, including chemistry, biology, materials science (such as crystallography, nanomaterials, etc.), physics, disaster resilience, cyberinfrastructure, communications, forensics, and others. NIST’s public data consist of carefully curated Standard Reference Data, legacy high valued data, and new research data publications. The repository is thus evolving both in content and features as the nature of research progresses. Implementation of the OAR infrastructure is key to NIST’s role in sharing high integrity reproducible research for measurement science in a rapidly changing world.

## Introduction

NIST research is predominantly characterized as “long tail” in terms of the data produced, i.e., small datasets that are highly varied in topic and content (Genova & Horstman 2016). This is colloquially described as “a mile wide and an inch deep” and may be classified as big data in context of variety and veracity. Newer, more modern laboratory instrumentation such as nuclear magnetic resonance spectrometers, electron microscopes, synchrotron beamlines, and high-performance computers usher NIST into the realm of managing the velocity and volume of big data. Furthermore, new strategic initiatives in the areas of artificial intelligence (AI) require an infrastructure designed to support digital mining and transformation. Management and exchange of the underlying research domain-specific data with both internal and external communities are important considerations for the OAR architecture and implementation.

The overarching goal of OAR is to deliver a robust research data infrastructure to share the results of NIST research with the community at large. Our strategy for achieving this goal involves collaborative data science as demonstrated through usage statistics from astronomical archives’ data discovery and access patterns (White et al. 2010). Organizations face many challenges striving to balance rapid advancements in technology and data driven research with internal operational costs and constraints. To meet these challenges, NIST assembled a diverse group of experts with key leaders and engaged stakeholders via cross-organizational advisors. This resulted in a joint effort to build an integrated system engineered to support data workflow processes, systems infrastructure, and public dissemination with secure publicly accessible platforms for scientific collaboration.

At the onset of the OAR project, priority was placed on developing a system that would allow us to comply with government open data policy (OMB M-13-13). This resulted in a baseline Minimum Viable Product (MVP), delivering a NIST public data listing (PDL) which enforces adherence to a new government data standard semantic model, the Project Open Data (POD) schema. The NIST PDL continues to be routinely harvested by the Department of Commerce and made available through the US data.gov web portal, which hosts records of all POD-compliant government public datasets. Following enactment of the OPEN Government Data Act (2019), updates and compliance of our OAR infrastructure will be further advanced.

**Figure 1:**
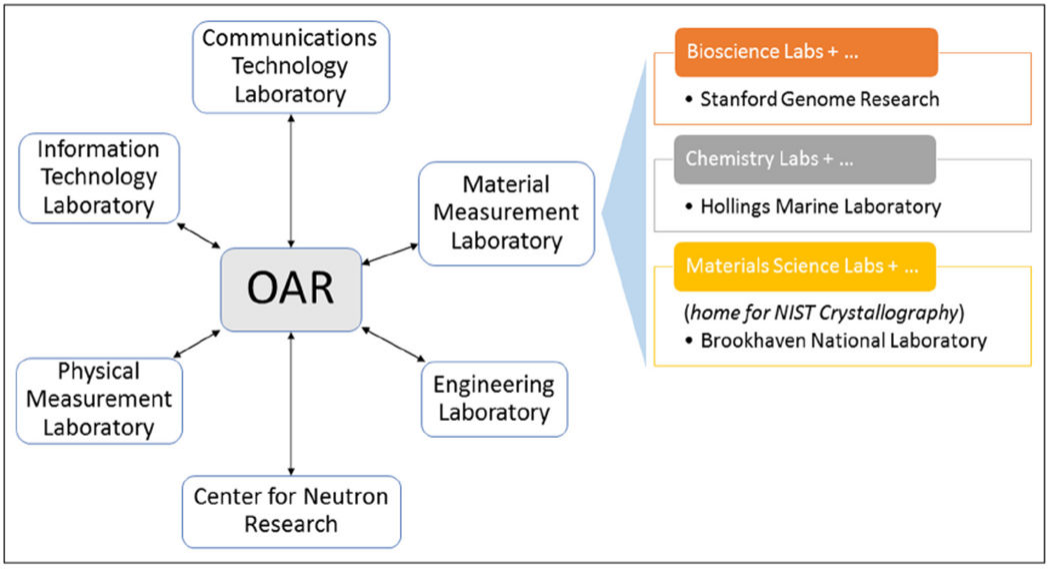
NIST OAR organizational context. NIST laboratory sites are in USA Gaithersburg, Maryland and Boulder, CO in addition to partner remote site locations as listed in the figure.

However, to achieve FAIR capabilities (Wilkinson et al. 2016), the OAR infrastructure supporting a science data portal and public data repository was designed to extend the limited MVP to include standard open formats, protocols, and demonstrated best practices in data management and publication to harness the full potential for community re-use of NIST research data products. The data portal provides both discovery and data access (distribution) capabilities through a science-oriented web user interface and REST (‘Representational state transfer’ 2019) application programming interfaces (APIs). The repository enables interoperability for scientific disciplines such as crystallography, biology, and chemistry as shown in the organization context (Figure 1) by supporting programmatic access to semantically rich data structures captured through the NIST data publication process. Key to the reuse of these data is the implementation of data citation for each of the records along with provenance metadata and link to usage policy.

## Architecture

The OAR architecture is in large part consistent with the consolidated Federal Enterprise Architecture (FEA, OMB 2013) reference models. FEA systems are fundamentally designed to identify common assets and shared technologies through a combination of enterprise class and open source solutions to ensure long term sustainability. In the case of OAR, FEA implementation was achieved through process models, data and logical workflows, application design and host infrastructure, architected to synergistically address stakeholder requirements. Adopting this robust architecture has demonstrated through iterative improvements in the OAR design, e.g. data review, usability features, that this model facilitates sustainability. Using agile methods, change may occur independently targeting different aspects of the FEA to streamline and modernize functionality. One realization with OAR maintenance is the risk associated with COTS enterprise solutions, i.e., budgeting funds for high license costs and rigidity in functionality, whereas the open source platforms are demonstrating benefit in the broader community context in keeping pace with evolving technologies especially in the areas of standard data semantic, syntactic, and schematic practices.

**Figure 2:**
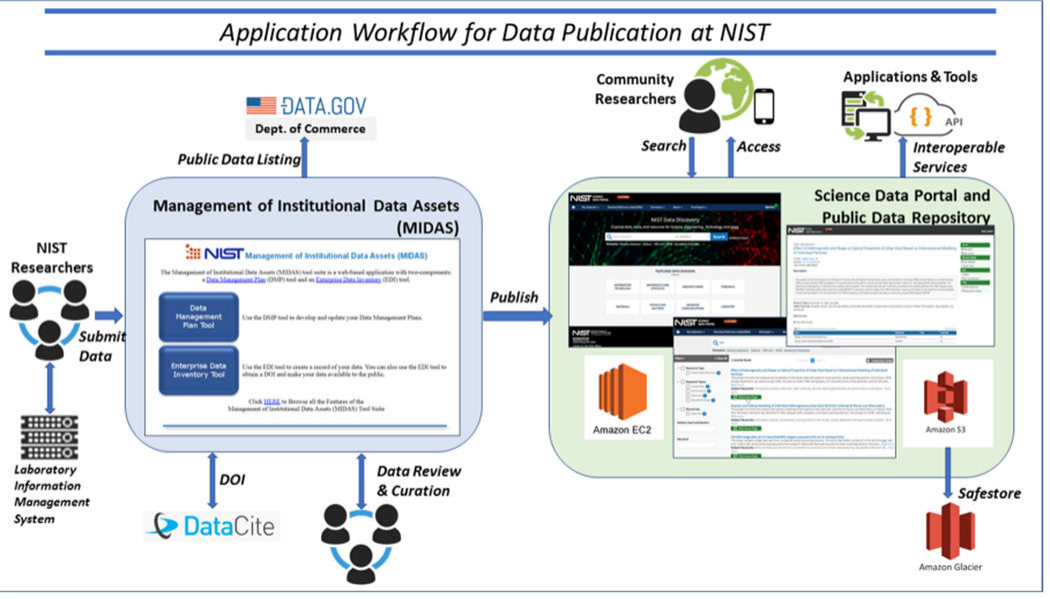
NIST data publication workflow showing external resource interfaces.

Figure 2 illustrates the high-level OAR application workflow for data publication. NIST researchers upload data products (files and metadata), which are generated from their Laboratory Information Management Systems (LIMS), to the OAR infrastructure via the NIST Management of Institutional Data Assets (MIDAS) tool. MIDAS also manages the data review process, and reporting/accountability for determining compliance with policies. Persistent identifiers are automatically assigned through a direct service interface to DataCite. Following approval from the review and curation processes, data are automatically preserved through a publishing service to the Public Data Repository (PDR) in a standard Bag1t format (Plante et al. 2019). The public repository datasets may subsequently be discovered through the Scientific Data Portal (SDP) on the NIST website (https://data.nist.gov). NIST has implemented the government-recommended cloud strategy (https://cloud.cio.gov/strategy/) as part of the OAR infrastructure, such that the OAR preserved datasets are hosted in a NIST Amazon Web Service (AWS) public enclave using the AWS Simple Cloud Storage Service (S3), and data are additionally copied to AWS Glacier storage as a long term “safestore”. The AWS Elastic Compute Cloud (EC2) server platform is used to host the repository and science data portal applications.

## OAR Implementation

The OAR project design was initiated in 2015 and completed a basic MVP release in 2017. The initial development phase involved a handful of dedicated staff which focused on production of the MVP and high priority features defined from inputs of the key stakeholders. As the system matured to include the described features in this article, additional shared staff resources have contributed to advance the capabilities for preservation, discovery and distribution services. Using an agile methodology, requirements are refactored into the system evolution based on testing and feedback collected from NIST science staff, organizational decision makers and external users.

**Figure 3:**
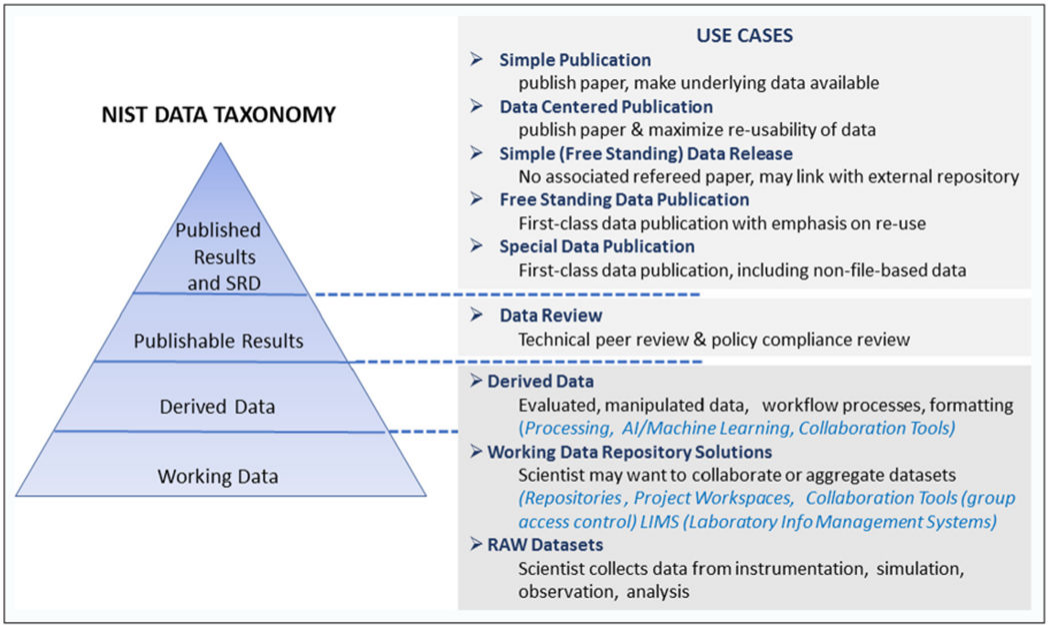
NIST data taxonomy pyramid for preservation, review, and discoverability requirements.

The OAR data portal and public data repository were implemented with a use case driven design approach. At the conceptual level, a data taxonomy was established as a guideline for preservation, review, and discoverability requirements for NIST data (Figure 3). This coarse hierarchy, mapped into common use cases for research data, serves to orient users how the OAR systems support the research lifecycle.

**Figure 4:**
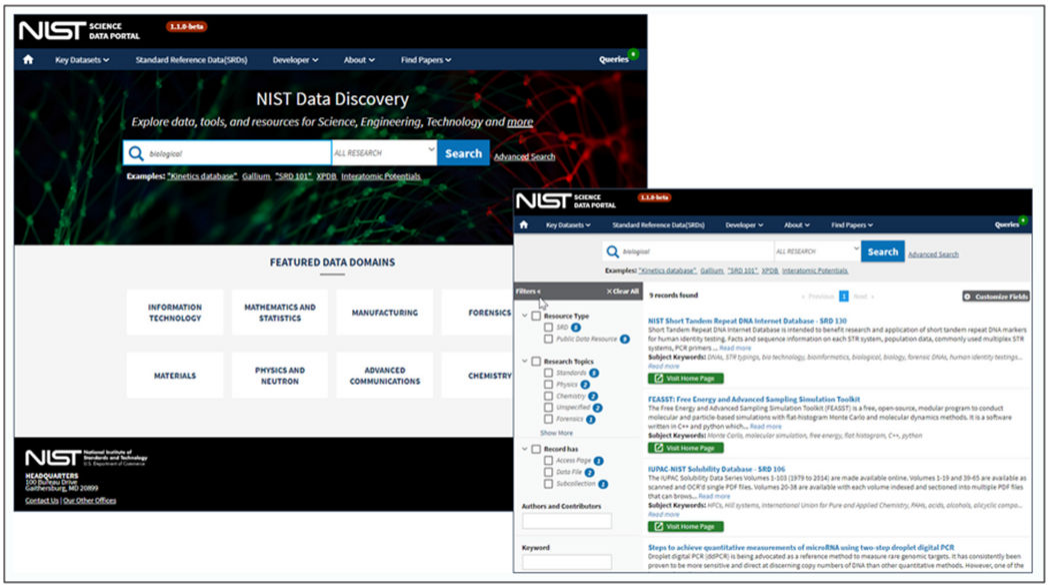
‘Search’ and ‘Search Results’ screenshots from the NIST OAR Science Data Portal homepage (https://data.nist.gov).

The OAR data publication workflow is composed of modular system components which include many best practices of data management systems such as review, curation, cataloging, indexing, preservation, discovery, filtering, and access. The data portal and public data repository applications are designed and developed using open source software as documented in public NIST OAR Github repositories (https://github.com/ usnistgov/oar-sdp & https://github.com/usnistgov/oar-pdr). As shown in Figure 4, the Science Data Portal (SDP) web user interface is customized to provide discovery and access to the NIST research domain datasets. The SDP homepage provides a search capability and menu links to NIST key datasets (including Standard Reference Data), developer information for software and APIs, and NIST manuscript publications.

**Figure 5:**
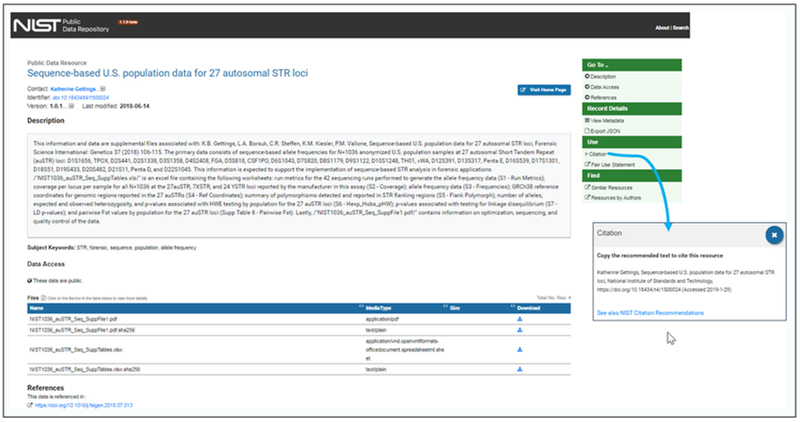
NIST Public Data Repository single record “landing page”, showing a citation available through linked navigation. The RESTful URL example is shown (https://data.nist.gov/od/id/6998B81EF78777B2E05324570681D4DC1911).

Search results are displayed with faceted filtering including categories for science research discipline, author, and record subcomponents. By selecting a record from the result list, a user is seamlessly navigated to a dynamically generated Public Data Repository (PDR) web “landing page” (Figure 5), a user-friendly presentation of the record metadata. The landing page URL is a RESTful endpoint and may serve as the resolved publication endpoint for the data DOI. The PDR landing page also includes a data access section for view and linked access to the data files’ distribution URLs. Figure 5 shows a Citation link, in the landing page’s right menu bar, to a preformatted citation for each resource that can be copied for reference to the data publication.

License and access rights are included in the record metadata and more broadly described in another link to the Fair Use Statement, also shown in Figure 5 just below the Citation. Since these data are public, there are no licensing restrictions.

## Domain Interoperability

The NIST Public Data Repository was designed to enable data interoperability as much as possible in order to maximize the usability of NIST data. To be usable, the data needs to tell its story in the language of not only the community it was intended for but also that of related communities that might apply it to cross-disciplinary research. This is particularly challenging for NIST data, given the broad spectrum of research domains that we serve. Nevertheless, we strove for a design that allows us to easily support common practices for interoperability, including (1) the export of metadata in multiple community formats and schemas, (2) support for community protocols for metadata harvesting, and (3) support for Google-like harvesting by embedding scrape-able metadata directly into dataset landing pages. Obviously, how we manage and share metadata is central to that interoperability.

We designed our repository’s internal metadata schema and format with an eye for interoperability—in particular, to make it easy to convert to different standard formats and support new formats and protocols over time. We refer to the schema as the NIST Extensible Resource Data Model (NERDm). NERDm metadata is formatted as JSON-LD (Sporny et al. 2014); this allows us to link every term in our schema to a concept in a documented community vocabulary or ontology. NERDm most closely resembles the POD schema which was designed for describing data resources provided by the US Government and which, naturally, we need to support. (POD itself is based heavily on the DCAT ontology (Maali & Erickson 2014)). Beside supporting the usual DCAT/Dublin Core concepts, the NERDm schema provides explicit places to include a variety of persistent identifiers, including DOIs, ORCIDs, and long-term URLs. The NERDm schema itself is defined using JSON schema (JSON Schema 2018); links to the schema documents as well as detailed documentation are available from our NERDm information page (https://data.nist.gov/od/dm/nerdm).

As its name suggests, extensibility is a key feature of NERDm and central in our strategy for enabling interoperability. There are three mechanisms for creating extensions of the core schema to support domain-specific metadata:

1. *Extensions that leverage linked data semantics*. NERDm supports a mechanism for plugging in schema extensions. This allows us to create extensions that support specific research domains. A metadata document formatted in JSON-LD includes a *context*—a built-in link to a kind of data dictionary—which relates metadata tags used in the document to predefined concepts. We use this mechanism to create extensions that can point to domain-specific ontologies. This allows generic linked-data clients to integrate metadata from different sources that refer to the same domain-specific concepts.
2. *Hooks for including domain-specific vocabulary terms*. The NERDm schema in various places includes a metadata tag named *topic*. It is a place to include descriptive keywords drawn from existing but arbitrary domain-specific vocabularies. Built into the *topic* element is an identifier for the vocabulary it was drawn from. Thus domain-specific vocabulary terms can be attached to individual files, data interfaces, or data collections as a whole.
3. *Leveraging external metadata types with JSON schema*. The NERDm extension framework allows us to make use of data types defined with JSON schema external to and independent of the NERDm schema. Using an enhanced JSON schema notation (Plante 2017), we can, in a NERDm metadata document, point to the location of the JSON schema that it conforms to. This allows us to embed directly terms from other JSON schema-based schemas (e.g. https://biocaddie.org/) directly into our metadata documents.

These techniques can be helpful to users and clients that might use our NERDm-formatted metadata directly; however, users are expected to be drawn more to the common formats of their own communities. The extension techniques are important to us internally because they make offering on-the-fly conversion to these formats easier; because the schema was assembled (and is being extended) from existing community concepts at the outset, we are not faced with an expensive “cross-walking” exercise to determine how to do the conversion. By supporting existing vocabularies and data types as directly as possible, it becomes easy to include metadata from multiple communities simultaneously, even if they cover the same, though variously nuanced, concepts.

## Conclusions

The implementation of the OAR scientific data infrastructure allows NIST scientists and professionals the means to share research data using standards and best practices adopted in the scientific community. This will help foster both national and international scientific collaborations, such as the Research Data Alliance and CODATA organizations along with industry and academic partnership. NIST’s leadership in metrology and standards will be more broadly distributed through the FAIR OAR infrastructure. We envision new data metrology methods will manifest for uncertainty and quality assessments aligning OAR with NIST objectives across discipline areas.

The initial release of the science data portal and public data repository has demonstrated value across NIST’s laboratories through reuse of open source solutions in congruence with securely managed government systems. Scientific staff have contributed positive feedback to these system capabilities which we attribute to striking a balance between what is required and personal benefit in giving credit and visibility to their research outputs. While there remains a learning curve for what it means to create high quality reusable data, we are seeing a steady increase in take-up from preliminary metrics. Data curation challenges are expected as these systems mature and NIST science data grows in capacity with modern instrumentation and theoretical computations output. We anticipate the OAR FAIR solution will facilitate the use of AI and machine learning applications and help solve many complexities in mining data rich resources. Natural language processing, semantic models, and algorithms will be simpler to build and execute across linked-data space. OAR is designed with a long-term vision to provide a rich infrastructure where creative data driven capabilities will foster new science. We must therefore collectively ensure our FAIR systems provide robust, reliable, and accurate data to maintain scientific integrity and safeguard our expertise.

## References

[R1] Genova F and Horstmann W 2016 Long Tail of Data – e-IRG Task Force Report. Available at URL http://e-irg.eu/documents/10920/238968/LongTailOfData2016.pdf [Accessed 29 Jan 2019],

[R2] Maali F and Erickson J 2014 Data Catalog Vocabulary (DCAT), W3C Recommendation Available at URL https://www.w3c.org/TR/vocab-dcat/ [Accessed 29 Jan 2019],

[R3] Office of Management and Budget. 2013 Federal Enterprise Architecture Framework version 2. Available at URL https://obamawhitehouse.archives.gov/sites/default/files/omb/assets/egov_docs/fea_v2.pdf [Accessed 29 Jan 2019],

[R4] Office of Management and Budget. 2013 M-13-13 Memorandum for the Heads of Executive Departments and Agencies. Available at URL https://project-open-data.cio.gov/policy-memo/ [Accessed 20 April 2019],

[R5] OPEN Government Data Act. 2019 Foundations for Evidence-Based Policymaking Act of 2017 Title II. Available at URL https://www.congress.gov/bill/115th-congress/house-bill/4174/text#toc-H8E449F-BAEFA34E45A6F1F20EFB13ED95 [Accessed 29 Jan 2019],

[R6] Plante R 2017 EjsonSchema. Available at URL https://github.com/usnistgov/ejsonschema [Accessed 20 Jan 2019],

[R7] Plante R, Greene G and Hanisch R 2019 The Baglt Packaging Standard for Interoperability and Preservation In: ADASS XXVIII ASP, San Francisco, CA, in press.

[R8] ‘Representational state transfer’. 2019 Wikipedia Available at URL: https://en.wikipedia.org/w/index.php?title=Representational_state_transfer&oldid=893532373 [Accessed 30 Apr 2019].

[R9] Sporny M, Longley D, Kellogg G, Lanthaler M and Linstrom N 2014 JSON-LD 1.0: a JSON-based Serialization for Linked Data, W3C Recommendation. Available at URL https://www.w3.org/TR/json-1d/ [Accessed 29 Jan 2019],

[R10] White R, 2010 The High Impact of Astronomical Data Archives. In: Astro2010: The Astronomy and Astrophysics Decadal Survey, Position Papers, no. 64.

[R11] Wilkinson M, 2016 The FAIR Guiding Principles for scientific data management and stewardship. Scientific Data, 3: 160018 DOI: 10.1038/sdata.2016.18 26978244 PMC4792175

